# Sex, War, and Disease: The Role of Parasite Infection on Weapon Development and Mating Success in a Horned Beetle (*Gnatocerus cornutus*)

**DOI:** 10.1371/journal.pone.0028690

**Published:** 2012-01-11

**Authors:** Jeffery P. Demuth, Amrita Naidu, Laura D. Mydlarz

**Affiliations:** Department of Biology, The University of Texas at Arlington Arlington, Texas United States of America; The University of Queensland, St. Lucia, Australia

## Abstract

While parasites and immunity are widely believed to play important roles in the evolution of male ornaments, their potential influence on systems where male weaponry is the object of sexual selection is poorly understood. We experimentally infect larval broad-horned flour beetles with a tapeworm and study the consequent effects on: 1) adult male morphology 2) male-male contests for mating opportunities, and 3) induction of the innate immune system. We find that infection significantly reduces adult male size in ways that are expected to reduce mating opportunities in nature. The sum of our morphological, competition, and immunological data indicate that during a life history stage where no new resources are acquired, males allocate their finite resources in a way that increases future mating potential.

## Introduction

Pressure on one sex to secure mating opportunities with the other has lead to some of the most extravagant and unusual phenotypes in nature, and consequently a great deal of attention from biologists. Often, sexually selected traits (SSTs) are male-specific because an inequity in parental investment makes females a limiting resource and increases the strength of selection on males in proportion to their variance in mating success [1], [2], [3], [4], [5]. Much of the history of sexual selection research can be summarized as an effort to find the primary factors influencing male mating success. In proximate terms the answer is simple; male mating success is determined either by female choice, resulting in selection for male ornaments, or by male-male contests, resulting in selection for male weapons, though some SSTs likely serve dual roles [6], [7], [8].

The majority of recent sexual selection literature emphasizes the evolution of male ornaments via female choice [9]. Much less attention has been devoted to the evolution of male weapons [10], perhaps because the direct utility of having a better weapon than ones competitor seems clear. However, theory developed explicitly to explain why ornaments should be reliable indicators of male quality [11], [12], also extends to the idea that not all males will benefit equally from producing the largest possible weapons [13], [14], [15]. For example, low quality males may waste energy investing in large weapons if they repeatedly lose contests to males whose large weapons are honest indicators of their high quality. One characteristic of male quality that SSTs are widely believed to indicate is parasite resistance. Since Hamilton and Zuk [16] initially proposed that brighter plumage indicates higher parasite resistance in birds, evidence for parasite mediated sexual selection for male ornaments has been observed in many taxa [4], [17], [18]. In stark contrast, the effect of parasites on male weapon development has rarely been tested [19], [20], [21], [22].

Arthropod systems present excellent models for testing parasite mediated sexual selection hypotheses. In particular, several studies demonstrate positive correlations between exaggeration of SSTs and immune function. For example, various measures of immune function are positively correlated with wingspot size in male damselflies [23], [24], [25], male song in crickets [26], [27], [28], and pheromone production in male beetles [29]. While these cases represent growing support for a connection between sexual selection and immunity, the evidence is not entirely consistent or comprehensive. For instance, studies that measure more than one aspect of immunocompetence (e.g. melanin encapsulation rate, and bacteriolytic activity) sometimes find that not all measures are correlated with exaggeration of the SST [20], [21], [24], indicating that different immune mechanisms may trade-off against each other [30]. Also, to directly assess whether immunocompetence is associated with SSTs while controlling for variation due to infection, studies often use immune system elicitors that do not require exposing animals to infective agents. These studies have a potential to miss aspects of infection that affect survival and/or mating probability in nature. For example, acanthocephalan parasites manipulate host behavior in ways that affect both survival and mating in many arthropod systems [31], [32]. Furthermore, only two studies test male structures used in combat (i.e. weapons) and these have mixed results. Rantala et al. [21] found that forceps size in earwigs is not indicative of immune function, but Pomfret and Knell [20] found that horn size in a dung beetle is an honest indicator. Our study aims to contribute to the understanding of parasite mediated sexual selection for weapons by investigating how active infection (i.e. infection with a live parasite) affects weapon development, immunity, and mate competition in a horned beetle.

The unsurpassed diversity of beetle horns, and the relative ease of rearing some species in laboratory conditions, provides an opportunity to further address the paucity of data relating parasites, immunity, and weapon development. Among the thousands of horned beetle species, many are known to use their horns as weapons in male-male contests to win access to females [10]. In all the cases that we are aware of, larger horned males win direct contests more often. Beetles are holometabolous, and as such many adult structures, including horns, undergo radical developmental growth and remodeling during a period where individuals do not feed (late larva and pupa stages; [33]. Given this revenue neutral environment, tradeoffs among weapons, other adult structures, and parasite defense may be particularly acute. Several, comparative and experimental studies have demonstrated such trade-offs between horn size and other morphological structures (e.g. antennae, eyes, wings, testes, and overall body size [34], [35], [36], [37], [38]; however, the only study connecting horn size and immune system investment showed that resource limitation during pupation does not affect a tradeoff with horn length [20].

**Figure 1:**
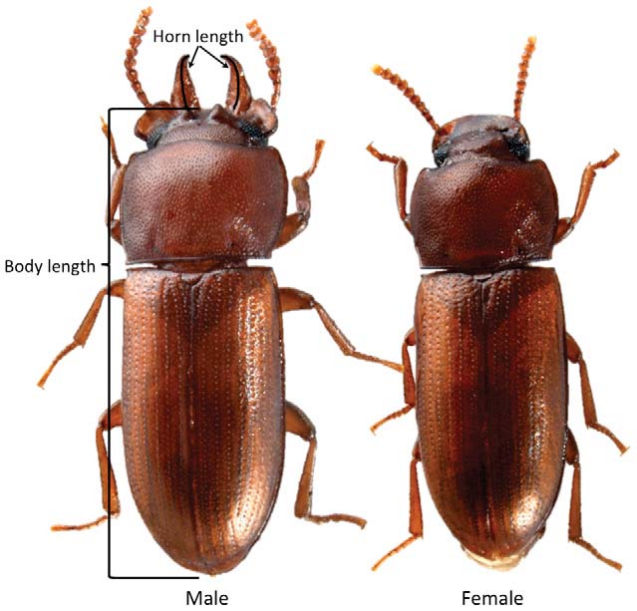
Male and female *Gnatocerus cornutus*. Reported body length is the average straight line distance from head to tip of elytra measured from dorsal and ventral views. Horn length is the average of right and left horns measured by a curved line along the middle of the horn viewed from the dorsal side.

Following, we present a study of the effect of parasite infection on weapon development, immune response, and mate competition in *Gnatocerus cornutus*, the broad-horned flour beetle. *G. cornutus* exhibits strong sexual dimorphism wherein males grow large mandibular horns, widened gena and a pair of small horns on the vertex of the head, but females completely lack these traits (Figure 1). Previous studies demonstrated that contests between rival males involve interlocking mandibular horns, pushing, lifting their opponent off the substrate, and/or biting [38], [39], [40]. Most often, larger males with larger mandibles win in combat; the gena and the head horns are not used [39]. In the present study we infect larval beetles with the rat tapeworm *Hymenolepis diminuta*. We then ask whether infection has an effect on: 1) male morphology, 2) immune response as measured by the circulating levels of pro-phenoloxidase (PPO) and phenoloxidase (PO), and 3) ability to acquire mating opportunities. Our fundamental hypothesis is that infected males will have smaller horns as both a direct consequence of infection and also because they divert resources from horn building to immune response. Given what is known about male-male contests in *G. cornutus* we also expect that if infection reduces male horn size, then infected males will have fewer mating opportunities. Additionally, the relative investment of infected beetles toward horn size or body size compared to uninfected individuals may inform us about the extent to which male-male combat is resolved by weapon size versus overall body size.

## Materials and Methods

### Beetle husbandry

The broad-horned flour beetle, *Gnatocerus cornutus* (Coleoptera: Tenebrionidae), is a cosmopolitan, secondary pest of stored products found predominantly in and around mill machinery where it feeds on milled grain [41]. *G. cornutus* likely has a tropical origin and is commonly found in warmer parts of the world or in temperate regions where it is protected from the cold [41]. Adult beetles measure 3.5–4.5 mm in length and are reddish-brown in color. Total development time (egg to adult) is approximately 47 days under our standard laboratory rearing conditions.

Except where indicated for particular experimental conditions, *G. cornutus* were reared on media consisting of 95∶5 ratio of whole-wheat organic flour∶brewer's yeast by weight. They were housed in a 24 h dark incubator maintaining 30°C and 70% relative humidity. Stock cultures are maintained in 45 L×30 W×8D cm covered plastic trays filled ∼3 centimeters deep with media. To initiate the present study we extracted pupae from stock cultures and transferred them to individual wells (3.5 cm diameter) of 24-well tissue culture plates (Cellstar; Greiner Bio-One, Germany). Gender was determined by presence of mandibular horns after eclosion. Virgin males and females from 15–30 days old were subsequently paired in glass vials 25Diam×95H mm containing standard media. Each pair was allowed to mate and lay eggs for one week, then transferred to fresh media, and following a second week of reproduction the parents were removed. Offspring from both weeks of egg laying were pooled at 2–3 weeks of age and then subdivided into 6-well tissue culture plates containing standard media. No more than 4 larvae were put in each well to limit density effects and cannibalism.

### Experimental infection with parasites

The rat tapeworm *Hymenolepis diminuta* is a cestode parasite of rodents and occurs in temperate zones worldwide. Adult tapeworms occur in the small intestine of the primary host and lay eggs that are excreted in rodent feces. The intermediate host, typically an arthropod, must consume the tapeworm eggs to initiate infection. Once ingested by the intermediate host, oncospheres hatch and penetrate the intestinal wall. Cysticercoid larvae develop within the hemal cavity where they live until the intermediate host is ingested by a new rodent host. To our knowledge, *H. diminuta* has not been reported to infect *G. cornutus*; however, it is able to use many arthropods as intermediate hosts and infection is well documented the family to which *G. cornutus* belongs (Tenebrionidae; e.g. [42], [43]. Infected rat feces for our study were collected by Dr. Sherman Hendrix (Gettysburg College) and shipped overnight to ensure maximum oncosphere survival and consequently maximize the probability that beetles exposed to the feces would become actively infected. The presence of *H. diminuta* eggs in rat feces was verified by examination under a dissecting microscope prior to use.

To initiate infection, late instar progeny were starved for 24 hours and then siblings from each mate pair were arbitrarily assigned to either the infection group or control group. For infection treatments, 0.3 g of rat feces were mixed with 0.2 g distilled water on 3.5 cm diameter circle of #3 Whatman filter paper and gently spread to cover the surface of the filter paper. The infected filter paper was then placed in a well of a 6-well tissue culture plate (Cellstar; Greiner Bio-One, Germany). Larvae from the infection group were then placed on the filter paper containing feces in each well. The number of larvae per well was limited to six. The control group was treated the same as the exposure treatment except that the filter paper was covered with 0.5 g of distilled water only. Both groups were maintained under these conditions for 48 hours and then transferred to new 6-well plates containing standard media where they matured under standard rearing conditions. In all cases there was clear evidence that larvae in the parasite exposure treatment had eaten infected feces. Because measuring immune response requires extracting all of the haemolymph from live beetles, we could not quantify infection and measure immune protein activity on the same individuals. Therefore, a subsample of individuals from six families were dissected as adults to estimate the infection rates among living beetles.

### Immune protein measurement

To measure the effect of infection on immune response we assayed for the melanin synthesis, or phenoloxidase (PO), cascade in adults from the infected and control groups. After the cuticle, the haemocytes and melanin synthesis pathway represents the first generalized responses against various immune challenges. Because PO is cytotoxic in its active state, it is constitutively found in the form of the zymogen prophenoloxidase (PPO) [44]. Upon infection, the parasite triggers localized proteolytic conversion of PPO into PO, which in turn catalyzes the oxidation of diphenols to quinones. Quinones and their radical intermediates are directly toxic to invading parasites and polymerize non-enzymatically to form insoluble melanin [45]. Ultimately, melanin and haemocyte encapsulation isolates and kills the invading parasite. In many, but not all, cases elevated PPO and PO activities are associated with increased ability to defend against infection [46], [47], [48], [49].

To measure PPO and PO, each individual was placed in a 1.5 ml microcentrifuge tube, dipped in liquid nitrogen for 5 seconds, and homogenized by grinding with a microcentrifuge Teflon pestle. 100 µl of beetle cell-lysis buffer (50 mM Tris-HCl pH 8.0, 10 µl/ml protease inhibitor cocktail containing 4-(2-aminoethyl) benzenesulfonyl fluoride (AEBSF), pepstatinA, E-64, bestatin, leupeptin, and aprotinin (Sigma-Aldrich, St. Louis, MO) 1 µM DTT and 160 µM EDTA was subsequently added and the cell homogenate and allowed to stand for 60 minutes on ice to extract the proteins. The resulting protein extract was centrifuged at 7000× g for 10 minutes, after which the supernatant was transferred to a new tube and kept on ice. To determine total protein concentration in each cell homogenate the Quick Start Bradford Protein Assay (Bio-Rad, Hercules, CA) was used following the manufacturers protocol. To determine constitutive PO activity 10 µl beetle protein extract was diluted with 65 µl 50 mM phosphate buffer pH 6.0 and 25 µl of 10 mM l-DOPA was added (final concentration of 2.5 mM l-DOPA). In order to evaluate the activity of PO that may have been stored as the pro-enzyme, the extract was treated with the serine protease, trypsin (bovine pancreas lyophilized powder, Sigma-Aldrich, St. Louis, MO) to promote the cleavage of the pro-enzyme PPO to PO; we are calling this measure PPO. 10 µl of beetle protein extract was diluted with 40 µl 50 mM phosphate buffer pH 6.0 and pre-treated with 0.0125 mg ml^−1^ trypsin dissolved in DI water for 30 minutes prior to addition of 25 µl of 10 mM l -DOPA (final concentration of 2.5 mM l-DOPA). Conversion of PPO to PO via exogenous trypsin exposure was successful as catalytic oxidation of l-DOPA to dopachrome was higher than untreated extracts. The assays were conducted in 96-well assay plates (Cellstar; Greiner Bio-One, Germany) and read on a Bio-Tek Synergy 2 with Gen5 software (Bio-Tek, Winooski, VT). The plates were read at time 0 and every 2.5 minutes for 30 minutes at 490 nm, although to confer to zero order kinetics we base reported calculations on the linear part of curve between 0 and 10 min. PO and PPO activity are represented as Abs_490 nm_ (Final_(10 min)_ – Initial_(0 min)_) mg protein^−1^ min^−1^.

### Competition experiments

Individual wells of 6-well tissue culture plates were used as arenas for all competition experiments. The bottom of each arena was covered with filter paper to provide traction and allow beetles to more easily right themselves if turned upside down. Filter paper was changed for each contest. All competition experiments included two males and one female. Behaviors were recorded with a Sony handycam HDR-SR5 video recorder inside an incubator illuminated with red light and maintained at 30°C and 70% relative humidity. The elytra of one of the males was marked with a small dot of Wite-Out® to aid in distinguishing between males during combat. Because copulation was impossible to determine definitively from our methods, a male's duration of contact with the female was recorded if contact lasted more than 5 seconds. Behaviors were scored for 30 minutes.

To test only the effects of beetle morphology on mate competition, we paired control group males (and female) using a random number generator (http://www.randomizer.org/). To test the effects of infection on mate competition we further attempted to control for genetic effects by pairing sibling males where one brother was from the infected group while the other was from the control group.

### Morphological measurements

To obtain measurements of body length and mandibular horn length (hereafter referred to as just “horn length”) adult beetles were immobilized, by anaesthetizing them with CO_2_ at 10 psi for 2 minutes, and then photographed under a Nikon SMZ 1500 dissecting microscope fitted with Nikon Digital Sight DS-Fi1 camera. We report body length as the average straight-line distance between the tip of the head and the tip of the elytra measured from both the dorsal and ventral surfaces. Horn length is the average of both right and left horns measured as a curved line along the median axis from the dorsal view (Figure 1). Length measures were computed using NIS-Elements image analysis software calibrated using a stage micrometer. Body mass was measured using an AT261 DeltaRange precision balance (Mettler-Toledo Inc., Columbus, OH).

### Statistical analyses

We analyzed horn size versus body length (or mass) measures for non-linear allometries following the steps outlined by Knell [50]. Briefly, we performed linear regression on log-log transformed and untransformed measurements, then plotted residuals from each to assess whether there was any systematic deviation from the linear prediction. While this informal inspection did not suggest any deviation from linearity, we also explored the potential for segmented or curvilinear relationships using the R package Segmented [51], and non-linear model fitting (see Results).

To test for the effects of parasite exposure on horn length and immune protein levels, we used generalized linear models (GLM) on log-transformed morphology measures with parasite treatment as a fixed factor and body length (and mass) as continuous explanatory variables. We included the parasite treatment×body size interaction term to test whether parasite exposure changes horn size to body size allometries.

To analyze mate competition trials, we performed paired t-tests to assess whether there was a significant difference in mean phenotypes between winners and losers, where winner was assigned to the male who spent more time in contact with the female. This addresses whether winners have larger horns (or other measure) than losers. We also performed Wilcoxon signed-rank tests to determine whether the number of seconds spent with the female is greater for larger males. We designated larger (or smaller) for each phenotype relative to the size of the directly competing males. The non-parametric test of ranks was used in this second analysis due to extreme, non-normal variation among times in different trials. This second analysis more directly addresses whether the phenotypic difference between competing males results in increased time spent with the female. Except where otherwise noted, statistical analyses were performed in JMP 9.0 (2010, SAS Institute Inc., Cary, NC) and considered significant at α = 0.05.

## Results

Dissection of live adult beetles indicated that our experimental infection protocol was highly effective. *Hymenolepis diminuta* cysticercoids were present in the haemolymph of 85.2% (23/27) of beetles. On average, each infected individual had 5 cysticercoids (4.91±0.72 s.e., range = 1–13). Dissections revealed cysticercoids to be clear or pale white ovals with elongated ends and darker centers representing the invaginated scolex. There was no visual evidence of melanin encapsulation by the host immune system under light microscopy.

In total we measured morphological and immune traits for 129 males from 39 families (mean number of sibs per family = 3.2; range 1–7). Family of origin had no effect in any of the statistical models. Consequently, subsequent analyses pool individuals across families. The lack of family effects may be a consequence of low within family sample sizes, but nevertheless indicates that our findings are not biased by relatedness or rearing group (which are completely coincident in our design).

### Tests for non-linear allometry

We found no evidence for non-linear allometries between horn size and body length or body mass. First, the distribution of control male horn sizes does not differ from a simple normal distribution (Shapiro-Wilk W = 0.987, p = 0.25) suggesting that multiple morphs are not naturally occurring in our sample. Second, inspection of residual plots from linear regression showed uniform scatter throughout the range of body lengths and masses, indicating that the straight line is satisfactory throughout the range of body sizes. Furthermore, using davies.test (implemented in the R package Segmented [51]) we did not find a significant breakpoint (or multiple breakpoints) to partition the relationship between horn length and body length (best at = 4461.5 mm, p-value = 0.665) or body mass (best at = 2.02 mg, p-value = 0.07). Since previous work indicated that a non-linear allometry exists in *G. cornutus*, we also performed a test similar to Okada et al. [39] for the presence of a quadratic relationship between body length and horn length. The nonlinear model *Y* = α_0_+α_1 *_ *X*+α_2 *_ *X* ^2^+*e*, where *Y* is ln(horn length) and *X* is ln(body length), yields a significant value for α_2_ (t = −2.44, p = 0.016) indicating nonlinearity similar to Okada et al. [39]. However, the likelihood ratio test indicates that the non-linear model does not describe our data better than the simple linear model *Y* = α_0_+α_1 *_ *X*+*e* (linear model: F = 208.38, r^2^ = 0.59, p = <0.0001; non-linear model: F_2,125_ = 110.77, r^2^ = 0.61, p = <0.0001; likelihood ratio *χ* ^2^ = 1.26, df = 1, p = 0.26). For the similar analysis using ln(body mass) instead of length also has a significant non-linear model (F_2,125_ = 122.21, r^2^ = 0.66, p = <0.0001) but in this case α_2_ is not significant (t = −1.15, p = 0.251).

### Effects of parasite exposure on morphology and immune response

**Figure 2:**
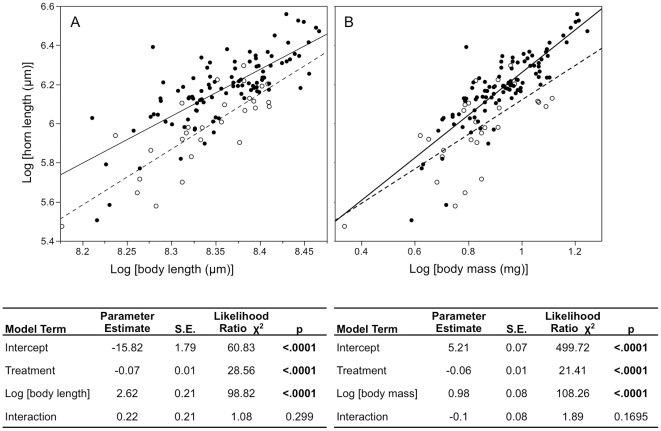
Effect of parasite exposure on male morphology. The relationships between horn size and (A) body length or (B) body mass plotted for exposed (open circles, dashed line) and control (closed circles, solid line) beetles. Below each plot we report the results of the GLM with p-values for significant model terms in bold. Both body size measures are significant predictors of horn size. Significant, negative treatment effects indicate that parasite infection reduces horn size. Non-significant interactions indicate no change in the slope of the body size to horn size allometry for length or mass.

Exposure to parasites significantly reduces horn length and body mass, but not body length (Table 1). Furthermore, the GLM including body length (or mass) as a covariate of horn length, indicates that parasite exposure significantly reduces horn size without affecting its allometry with other body size measures (i.e. the slope is unaffected by exposure, Figure 2).

**Table 1:** Effect of parasite exposure on beetle morphology and activation of the melanin-synthesis cascade.

	control	exposed	effect		
	(n = 100)	(n = 28)	(%)	t	p-value
Horn length (µm)	489.92±8.25	401.42±14.63	18.1	5.27	**<0.001**
Body length (µm)	4277.32±24	4186.21±45.54	2.1	1.77	0.084
Body mass (mg)	2.55±0.035	2.34±0.069	8.2	2.76	**0.009**
PPO/mg total protein	1.172±0.032	1.212±0.084	−3.3	−0.44	0.667
PO/mg total protein	0.068±0.002	0.081±0.011	−19.1	−1.08	0.306

Mean ± s.e. are reported for each measure. % Effect = (1−[exposed/control])*100.

The analysis of PPO and PO activity suggests that these immune proteins are unaffected by *H. diminuta* infection, body length, mass, horn length, or any interactions among these factors (Table 2). While somewhat surprising to us, the lack of significant difference in PPO or PO activity between exposed and control beetles is consistent with our observation in dissected beetles that *H. diminuta* does not trigger the melanization cascade (i.e. cysticercoids were not encapsulated). Despite the lack of change in PPO or PO, the high infection frequency among dissected beetles and the significant morphological differences between exposed and control beetles suggest that it is reasonable to infer that the vast majority of exposed individuals were indeed infected.

**Table 2:** Main effects of parasite exposure treatment, body size and horn size on PPO and PO activity.

	PPO activity				PO activity			
	Parameter		Likelihood		Parameter		Likelihood	
Model Term	Estimate	S.E.	Ratio χ2	p	Estimate	S.E.	Ratio χ2	p
Intercept	−0.16	6.877	0.001	0.982	0.378	0.61	0.384	0.535
Treatment	0.063	0.165	0.145	0.704	0.023	0.015	2.524	0.112
BodyMass	0.181	0.584	0.096	0.757	−0.002	0.052	0.002	0.969
BodyLength	<0.001	0.001	0.153	0.696	<0.001	<0.001	0.742	0.389
HornLength	0.005	0.006	0.701	0.403	<0.001	0.001	0.03	0.862

Fully factorial GLM also indicated no significant interaction among these main effects. Degrees of freedom = 1 in all cases (i.e. testing the full model versus the one with the designated effect removed).

**Figure 3:**
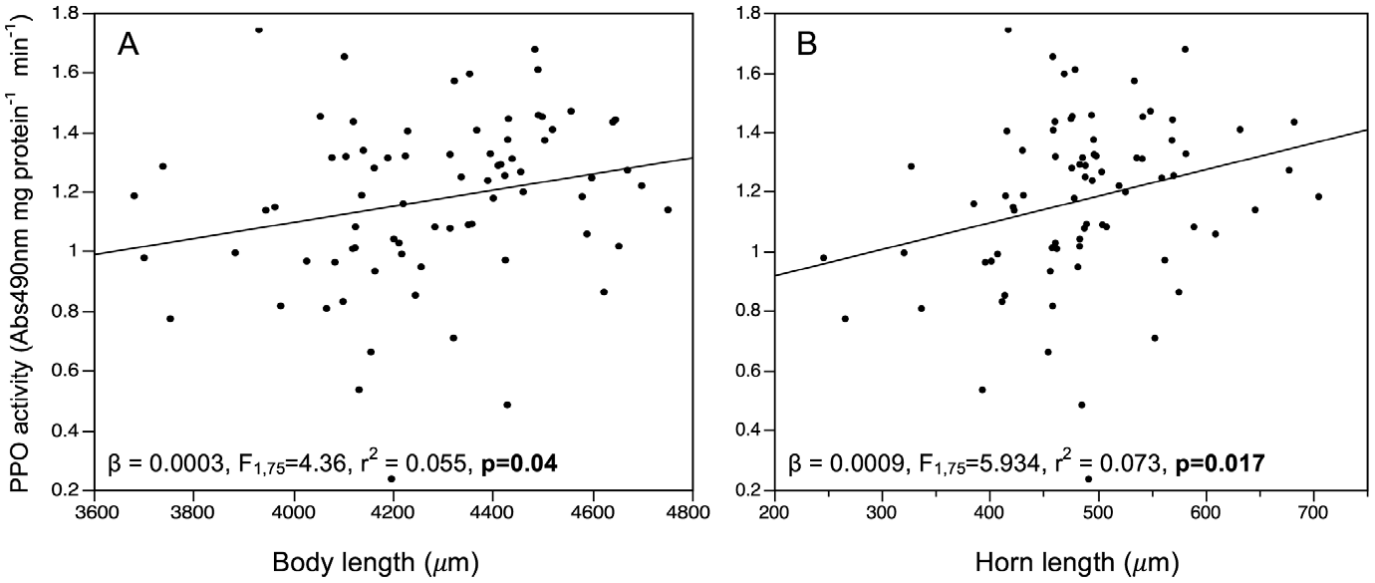
PPO activity is positively related to body length and horn length in control beetles. (A) Body length and (B) horn length are significant predictors of PPO activity in control beetles. Horn size is not significant after accounting for the contribution of body size (see text).

Additional analysis of PPO and PO activity within exposed and control beetles found a significant positive relationship of PPO with both body and horn length in control beetles (Figure 3). This relationship did not hold for exposed beetles or when horn length is corrected for body size (e.g. using residuals of the body size vs horn size regression).

### Mate competition

We performed a total of 29 mate competition trials. Males were frequently seen interlocking their horns and pushing each other around the arena. Some males were seen lifting their rivals off the substrate with their mandibles. These observations of male-male fighting behavior are consistent with previous work [38], [39] and we also observed a decrease in aggressiveness of losing males [40]. We did not witness male attacks against the female in our trials. Because our study included females within the competitive arena we were able to observe that losing translates into nearly complete exclusion from mating opportunities. After their retreat from the immediate location of the fight, losing males in our study typically became much more sedentary and had only incidental contact with the female as she explored the arena. Consequently, there were few observations of one male directly interfering with another male's mating opportunity. Winning males continued to be active, exploring the arena, courting, and mating. After mating, the winning male and the female explored the arena independently, but repeated matings between the winning male and female were common. Marking with Wite-Out® had no detectable effect on the outcome of competition (marked male won 15 and lost 14; Wilcoxon signed-ranks test for ranked time grouped by marked and not marked yields; S = 31.5, p = 0.505).

Early mortality among beetles in the exposed treatment resulted in only seven mate competition experiments pairing control and exposed beetles. This limited sample did not show evidence that parasite exposure (and presumably infection) directly affected the outcome of competition. Wilcoxon signed-rank test showed no difference in time spent with females between exposed and control males (S = 2.0, p = 0.813) with control males winning (i.e. having more mating time) in only 57% (4 of 7) contests. However, the conclusion that there is no effect of infection should be tempered by the small sample and because the effects of horn size and body size are confounded with parasite exposure treatment in several cases (e.g. the control beetle was also the larger horned beetle in 4 cases). Since infection did not statistically impact the winner of mate competition in the above trials, for subsequent analyses we pooled all of the contest data to assess the effects of morphology on the outcome of competition (note that excluding trials with exposed beetles does not alter significance of any tests but increases the s.e. reported in Tables 1 and 3).

**Table 3:** Results of male-male competition experiments.

					bMedian time in seconds			
	aMean phenotype ± s.e.				(25%–75% quantiles)			
Trait	loser	winner	*t*	p	smaller	larger	*S*	p
Horn length	474.6 µm	526.8 µm	2.53	**0.017**	39	104	62.5	0.181
	±15.7	±14.5			(17.5–165.5)	(26–246)		
Body length	4248.3 µm	4364.4 µm	2.47	**0.02**	38	123	106.5	**0.018**
	±37.7	±44.2			(17–86.5)	(26–252)		
Relative horn size^c^	−12	12	−1.22	0.233	38	80	61.5	0.188
	±12.3	±11.3			(17.5–165.5)	(28–270.5)		
Body mass	2.5 mg	2.57 mg	0.79	0.218	38	80	51	**0.04**
	±0.05	±0.07			(17–104)	(21–289)		

a - Paired t-test results for mean phenotype of winners versus losers. Winner is defined as the male who spent the most cumulative time in contact with the female.

b - Wilcoxon signed rank tests for smaller versus larger male's time spent with female. Smaller and larger were defined independently for each pair of competing males. Significant p-values are in bold throughout. n = 29 except for body mass where n = 19.

c - residual of horn length regressed on body length.

Winning beetles had significantly larger horns, and longer bodies, but did not have relatively larger horns after correcting for body size differences, and were not significantly heavier than their rivals (Table 3). Having a longer body is a slightly better predictor of victory (21/29 = 72.4%) than longer horns (19/29 = 65%). In several cases where shorter bodied (or shorter horned) males lost, horn and body size were discordant between rivals (i.e. smaller body but a larger horns than the rival). Our alternative analysis of whether male morphology translates into more time with the female reveals that only body length (*S* = 106.5, p = 0.009) and mass (*S* = 51, p = 0.02) significantly impact the duration (Table 3). This further highlights that horn size appears to be less important than body size in determining the outcome of competition.

## Discussion

While parasites and immunity are widely believed to play an important role in mate choice and the evolution of male ornaments [16], [17], [52], [53], their potential impact on sexual selection involving male weaponry is poorly understood [10], [19], [20]. Our study examines the effect of *Hymenolepis diminuta* infection on male morphology, immune protein level, and mate competition. Our data show that infection reduces male size in a way that is likely to reduce mating opportunities. However, whether the size reduction reflects a trade-off with immunity and whether infection directly impacts males' competitive ability (i.e. not through the effect on morphology) remain obscure.

### Infection induced morphological changes may mediate mating opportunities

**Figure 4:**
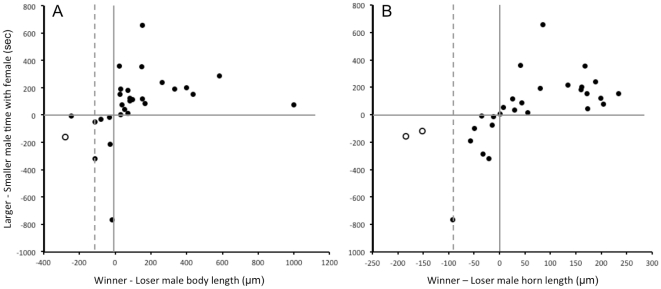
Differences in time spent with the female as a function of difference in (A) body length, and (B) horn length. Solid lines mark zero difference in time and size. In the upper right quadrant larger males spend more time with females (as expected), and in the lower left, smaller males spend more time (counter to expectation). Dashed lines mark the difference between the average size of uninfected and infected males (Table 1). Males are expected to win only 3.4% of the time (1 of 29) when their body length disadvantage exceeds the average reduction due to infection - or 6.9% (2 of 29) for average infection induced horn length reduction (open circles).

Our results indicate that infection causes a much larger reduction in horn size than body size (Table 1, Figure 2). Howard and Minchalla [19] proposed that when weapons used exclusively in mate competition are energetically expensive to produce, if they are reduced less than body size in response to infection, then it suggests that weapon size is likely to be more important than body size to determining the outcome of competition; but conversely, if weapons are reduced more than body size, then weapon size is likely to be less important than body size to determining the outcome of competition. Thus disproportionate reduction in weapon investment by *G. cornutus* males predicts that horn size is less important than body size to the outcome of male-male competition. This prediction is directly supported by our competition experiments where the relative horn length of male *G. cornutus* (after correcting for body size) does not differ significantly between winners and losers, nor does it impact the amount of time spent with females (Table 3). Our results demonstrate that male beetles invest finite resources in a way that increases their opportunity to mate even though the magnitude of horn and body size reduction in our experiments suggests that few if any infected males would win in nature when competing against males who are resistant to infection (Figure 4).

The morphological changes induced by parasite infection are similar to those found in response to artificial selection for increased or decreased horn size. Specifically, the allometry between horn size and body size remained constant among selection lines [38] the same way that it remained constant between infected and control groups in our study. In concert these data suggest that *G. cornutus* experiences selection for a ratio of horn size and body size rather than simply one or the other. Interestingly, artificial selection on male horn size causes negatively correlated responses in testis size, ejaculate investment, and female fitness (e.g. selection for larger horns produces males with smaller testis, lower ejaculate investment [54], and female siblings that have lower lifetime reproductive success [55]). Furthermore, if *G. cornutus* males that lose a contest are subsequently paired individually with a female and allowed to mate, on average they transfer more sperm than winners [56]. So smaller males who lose fights may have an alternative strategy to increase reproductive success via sperm competition [54], [56]. In the future it would be interesting to investigate whether the optimal investment we see for body size versus horn size also extends to the wider set of evolutionary tradeoffs observed in selection studies.

### Innate immune response to Hymenolepis diminuta infection

While infection clearly affects changes in *G. cornutus* morphology, we did not see the anticipated effect of infection on circulating immune protein levels. We remain cautious about interpreting this finding however. Pomfret and Knell [20] showed that PO and encapsulation activity increased significantly during maturation from freshly emerged to one week old adults, in well fed *Euoniticellus intermedius* (a horned Scarabid beetle). In our study, resources acquired between infection and our immune protein sampling (approximately two weeks) might obscure differences in immune proteins that were present during critical stages of development. Alternatively, our observation that cysticercoids had no melanization in our dissected beetles suggests that infection with tapeworm eggs simply does not initiate the melanin-synthesis cascade in *G. cornutus*.

Studies using the confused flour beetle, *Tribolium confusum*, and mealworm *Tenebrio molitor* (two other Tenebrionid beetles) demonstrate that response to *Hymenolepis* infection can vary among host and parasite species. For instance, in *T. confusum*, *H. diminuta* elicited a milder response than *H. citelli* and the host response to *H. diminuta* was generally ineffective and short lived (3 to 5 days in *T. confusum*, which is less than the time from infection to maturation in our study). Importantly, stronger immune reactions occurred in cases where *Tribolium* was not a natural intermediate host for the infecting species of *Hymenolepis*. We know of no previous work on infection and immunity in *G. cornutus*, but if the findings from *Tribolium* can be generalized, the lack of immune protein response and failure to eliminate the infection may suggest that *H. diminuta* is a natural parasite of *G. cornutus*. Although, infected beetles in our study did experience higher adult mortality (72%) than seems likely for a natural parasite, the high mortality may be a consequence of our protocol, which aimed to assure infection and as a side effect might have also resulted in higher than normal initial parasite loads.

In *T. molitor*, *H. diminuta*'s effects on host behavior, reproduction and longevity have been well studied. Hurd and colleagues have shown that infected females suffer a costs to egg viability [57]. Also, infection causes enlargement of male accessory glands and may result in increased offspring number when mated to uninfected females [58]. Infected *T. molitor* demonstrate stereotypical sick behaviors such as decreased activity and decreased photophobia [59], but they live longer than controls [60]. The potential of *H. diminuta* infection to increase the quality of *G. cornutus* male ejaculates is particularly interesting in light of the potential synergistic effects of increased sperm deposition by loser males [56]. However, there are clear differences between infection effects between *Tenebrio* and *Gnatocerus* hosts. For instance, infected *T. molitor* have increased young adult survivorship, the opposite of what we see in *G cornutus*. The mortality difference may be a consequence of relative size, as *Tenebrio* is orders of magnitude larger than *Gnatocerus*, but cautions against cross species generalization.

### The role of immunocompetence in male-male contest competition

Even though *H. diminuta* infection did not induce PPO, PO or visible melanization in *G. cornutus*, we did find a weak but positive relationship between body length, horn size and PPO in healthy, control males. Consistent with our finding that body size is a better predictor of male mating success than relative horn size, body size was a better indicator of immune protein level than relative horn size. To the extent that circulating levels of PPO are indicative of an individual's ability to fight infection (via conversion of the zymogen PPO to active PO upon signaling) the combination of larger males having higher immune protein activity and increased mating opportunities is consistent with indicator models of sexual selection. The trait that is the best predictor of an individual's mating opportunities is also the best indicator of their immune function.

These results inform a more specific version of parasite mediated sexual selection that attempts to specify the physiological currency mediating tradeoffs with SSTs, the immunocompetence handicap hypothesis (ICHH). ICHH has been formulated in several ways beginning with Folstad and Carter's [53] observation that testosterone increases SST expression while simultaneously depressing immune function. Given the lack of a testosterone homolog in invertebrates, general energy budget [61], [62], juvenile hormone [63], and oxidative stress due to activation of the PPO cascade [64], [65] have all been proposed to play analogous roles in mediating the tradeoff between SSTs and immunity. Morphological changes in the absence of PPO or PO changes seen in our study, are not consistent with the ICHH. However, we cannot exclude the possibility that juvenile hormone may affect a tradeoff with other mechanisms of immune response including opsonization, phagocytosis, coagulation, production of antimicrobial peptides, and a range of other defense molecules (e.g. lysozyme, and proteolytic and hydrolytic enzymes) [66]. *Hymenolepis* infection does briefly impact juvenile hormone levels in *T. molitor* adults [67], but its effect on earlier developmental stages, when SST development is expected to occur, is unknown. If occurring, juvenile hormone dependent immune responses are ineffective (at least against *H. diminuta*) given the high mortality and persistence of infection into *G. cornutus* adulthood.

While studying weapon development in beetles with and without active infection provides a clearer picture of the consequent morphological and mate competition costs to males in nature, it also limits the scope of our interpretation. At least three important avenues for future investigation are clear from the present study. First, infection and immune response may have different consequences at different developmental time points. For instance, infection at early larval stages may have no net effect on weapon development if individuals can recover from infection and delay pupation until obtaining equivalent resources to uninfected individuals. Second, future studies should test a broader set of infectious agents and potential immune responses to see whether the similar consequences to morphology emerge. Finally, the utility of immune system elicitors such as bacterial lipopolysaccharides is that they do not confound 1) the physiological cost of mounting an immune response, with 2) direct damage from the infective agent. To better understand the currency mediating morphological, behavioral and physiological tradeoffs with weapons development, future studies will benefit from knowing the relative contributions of these two components in *G. cornutus*.
